# Clinical outcomes and predictive factors for failure with MPFL reconstruction combined with tibial tubercle osteotomy and lateral retinacular release for recurrent patellar instability

**DOI:** 10.1186/s12891-021-04508-x

**Published:** 2021-07-21

**Authors:** Peng Su, Xiumin Liu, Nengri Jian, Jian Li, Weili Fu

**Affiliations:** 1 Department of Orthopaedic Surgery, West China Hospital, Sichuan University, 37 Guoxue lane, Wuhou District, Chengdu, China; 2 Department of Radiology, West China Hospital, Sichuan University, 37 Guoxue Lane, Wuhou District, Chengdu, China

**Keywords:** Medial patellofemoral ligament reconstruction, Tibial tubercle osteotomy, Lateral retinacular release, Patellar alta, Recurrent patellar instability, Caton-Deschamps index, Tibial tubercle–trochlear groove distance, Trochlear dysplasia

## Abstract

**Background:**

Medial patellofemoral ligament (MPFL) reconstruction combined with tibial tubercle osteotomy (TTO) and lateral retinacular release (LRR) is one of the main treatment methods for patellar instability. So far, few studies have evaluated the clinical effectiveness and assessed potential risk factors for recurrent patellar instability.

**Purpose:**

To report the clinical outcomes of MPFL reconstruction combined with TTO and LRR at least three years after operation and to identify potential risk factors for recurrent patellar instability.

**Methods:**

A retrospective analysis of medical records for patients treated with MPFL, TTO and LRR from 2013 to 2017 was performed. Preoperative assessment for imaging examination included trochlear dysplasia according to Dejour classification, patella alta with the Caton-Deschamps index (CDI), tibial tubercle–trochlear groove distance. Postoperative assessment for knee function included Kujala, IKDC and Tegner scores. Failure rate which was defined by a postoperative dislocation was also reported.

**Results:**

A total of 108 knees in 98 patients were included in the study. The mean age at operation was 19.2 ± 6.1 years (range, 13–40 years), and the mean follow-up was 61.3 ± 15.4 months (range, 36–92 months). All patients included had trochlear dysplasia (A, 24%; B, 17%; C, 35%; D, 24%), and 67% had patellar alta. The mean postoperative scores of Tegner, Kujala and IKDC were 5.3 ± 1.3 (2–8), 90.5 ± 15.5 (24–100) and 72.7 ± 12.1 (26–86). Postoperative dislocation happened in 6 patients (5.6%). Female gender was a risk factor for lower IKDC (70.7 vs 78.1, *P* = 0.006), Tegner (5.1 vs 6.0, *P* = 0.006) and Kujala (88.2 vs 96.6, *P* = 0.008). Age (*p* = 0.011) and trochlear dysplasia (*p* = 0.016) were considered to be two failure factors for MPFL combined with TTO and LRR.

**Conclusion:**

As a surgical method, MPFL combined with TTO and LRR would be a reliable choice with a low failure rate (5.6%). Female gender was a risk factor for worse postoperative outcomes. Preoperative failure risk factors in this study were age and trochlear dysplasia.

**Level of Evidence:**

Level IV; Case series

## 1. What is known about this subject?

Recurrent patellar instability is a common problem, which could cause anterior knee pain, limit the motion of the knee joint, and increase the risk of patellar osteoarthritis. The stability of patellofemoral joint is maintained by the joint action of bony anatomical structure and soft tissue on patella. A few studies have proved that the surgical procedures—MPFL alone or MPFL combined with TTO—can get good results. However few studies described the good outcome of MPFL reconstruction combined with LRR and TTO, information about multiple risk factors for poor outcomes of the combined treatment is rare, and no articles reported risk factors for failure of the combined treatment.

## 2. What this study adds to existing knowledge

As a surgical method, MPFL combined with TTO and LRR would be a reliable choice with a low failure rate. Female gender was a risk factor for worse postoperative outcomes. Preoperative failure risk factors in this study were age and trochlear dysplasia.

## Background

Recurrent patellar instability is a common problem, which could cause patellofemoral pain, limit the motion of the knee joint, and increase the risk of patellofemoral osteoarthritis [1]. The stability of patellofemoral joint is maintained by the joint action of bony anatomical structure and soft tissue on patella.

The medial patellofemoral ligament (MPFL) is the most important soft tissue to keep patellar from lateral displacement from zero to thirty of knee flexion. It has been proved that the injury of MPFL happens during all lateral patellar dislocations [2, 3]. With the injury of MPFL, tension and contracture of lateral retinaculum always happen. Over-constraint by the lateral retinaculum is one of the causes of patellofemoral disorders, particularly pain in the patellofemoral joint, patellofemoral instability and chondromalacia of the articular cartilage of the patellofemoral joint. Therefore, MPFL reconstruction and lateral retinacular release (LRR) have become popular in the surgery for patellofemoral instability. However, patellar instability is a multifactorial problem [4], and in some cases, it could be necessary to combine MPFL reconstruction and LRR with other surgical procedures, which include bony procedures, such as distal and/or medial transfer of the anterior tibial tubercle and trochleoplasty.

Tibial tuberosity to trochlear groove (TT–TG) distance is an index for measuring the lateralization of the tibial tuberosity [5]. Except for TT-TG distance, tibial tuberosity to posterior cruciate ligament (TT-PCL) is also an ideal index for measuring the lateralization, which is not influenced by knee rotation. But numerous studies proved that the mean differences in the TT-PCL value between the control and dislocation groups were both 2 to 3 mm (smaller than MCID-5 mm), which meant that TT-PCL was not clinically meaningful. Finally, TT-TG was used for measuring the lateralization of tibial tuberosity. TT–TG distance more than 20 mm has been suggested as an indication for tubercle medialization with a tibial tubercle osteotomy [5, 6]. And Caton-Deschamps Index (CDI) more than 1.2 is an index for defining patella alta [7]. Patella alta can be corrected by the distalization of tibial tubercle. Trochlear dysplasia is common among patients with recurrent patellar instability. Trochleoplasty is recommended in case of severe trochlear dysplasia [8–10]. However, current clinical results demonstrate only fair outcomes and a high incidence of arthritis at long-term follow-up [11, 12].

So far, numerous studies described the good outcome of MPFL reconstruction alone or MPFL reconstruction with tibial tubercle osteotomy (TTO) [7, 13], but whether LRR was performed at the same time and the indications were not clear, information about multiple risk factors for poor outcomes of combined treatment of MPFL reconstruction, TTO and LRR together was rare, and few articles reported risk factors for failure of combined treatment. The purpose of this study was to report the outcomes of combined treatment and assess the potential risk factors for poor outcomes and failure (postoperative dislocation).

## Methods

This study received institutional review board approval. All procedures were performed in accordance with relevant guidelines.

### Patients

**Fig. 1:**
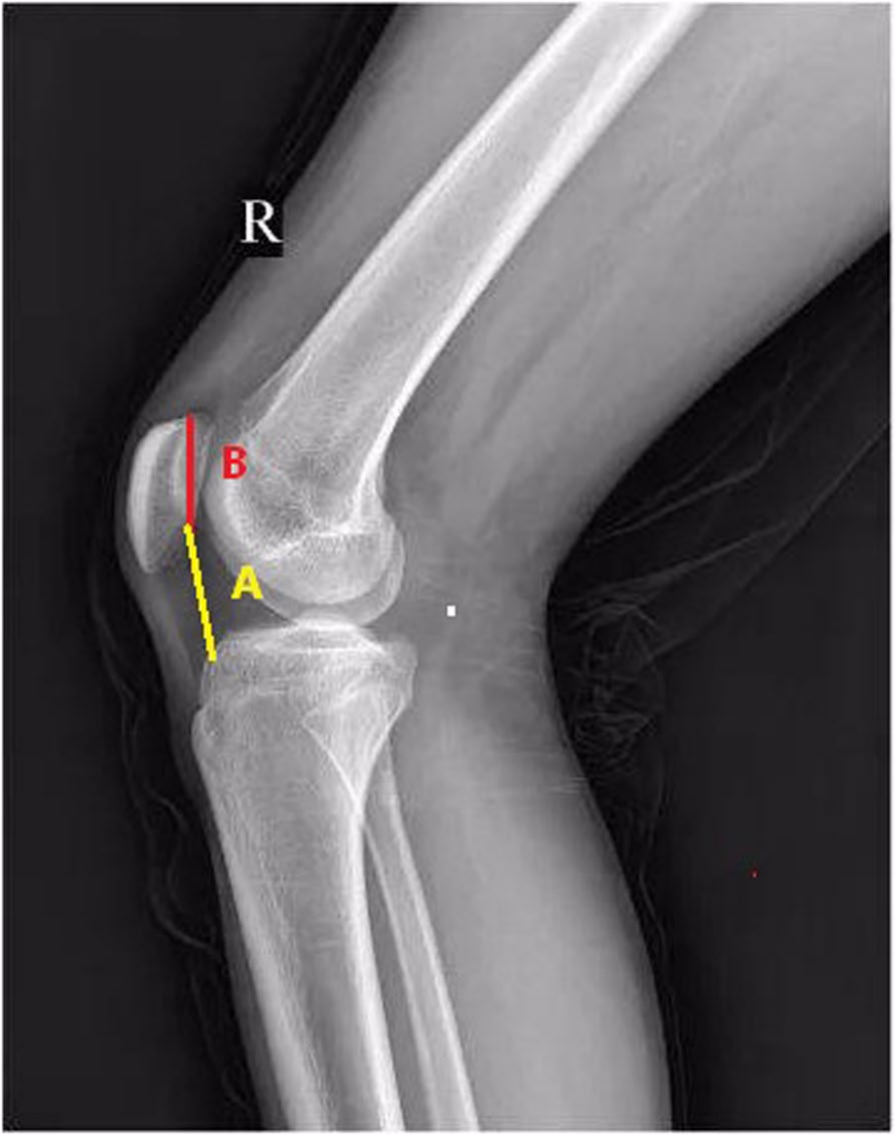
Line A (yellow line): the distance from the lowest point of patellar-articular surface to the anterior edge of tibial plateau; Line B (red line): the length of patellar articular surface. CDI = A/B; CDI_normal_ = 1; A_normal_ = B; A_actual_ = CDI_actual_ × B; The amount of distalization = A_normal_-A_actual_

**Fig. 2:**
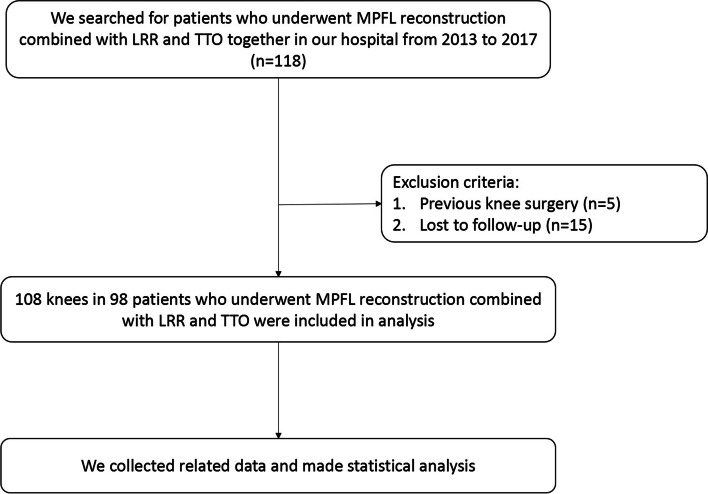
The Flow Chart of the Research. It mainly introduced the process of screening patients

The patients included in the study had at least 2 patellar dislocations. One experienced surgeon (JL) performed MPFL reconstruction combined with LRR and TTO treatment from 2013 to 2017. The indications for TTO were CDI ≥ 1.3 (transfer distally; The normal index of CDI is 1.0, the amount of distalization is A_normal_-A_actual_; Fig. 1) and TT-TG ≥ 20 mm (transfer medially, the amount of medialization is at least 10 mm, make sure that the distance is less than 20 mm after osteotomy). The patella was pushed toward the medial side. If the patella moved less than one fourth of the patella, the lateral retinaculum was released. A retrospective analysis of collected data from the authors’ institution was performed, and all patients who had patellar instability were included and underwent combined treatment. The patients with previous knee surgery were excluded. The details of screening patients were shown in Fig. 2.

### Clinical and Radiological Assessment

The records were reviewed for preoperative data including the age at the time of surgery, body mass index (BMI), gender, and follow-up time. Preoperative radiographs were reviewed for patellar alta and trochlear dysplasia. Patellar alta was defined using a Caton-Deschamps Index (CDI) of more than 1.3 based on lateral radiographs. Trochlear dysplasia was defined using Dejour classification based on computed tomography (CT) slices. Preoperative TT-TG distance was defined based on CT slices. Measurements were performed by 1 musculoskeletal radiologist. Postoperative indicators included postoperative re-dislocation besides Tegner, IKDC and Kujala scores. The shortest postoperative follow-up time was three years.

### Surgical Procedure

**Fig. 3:**
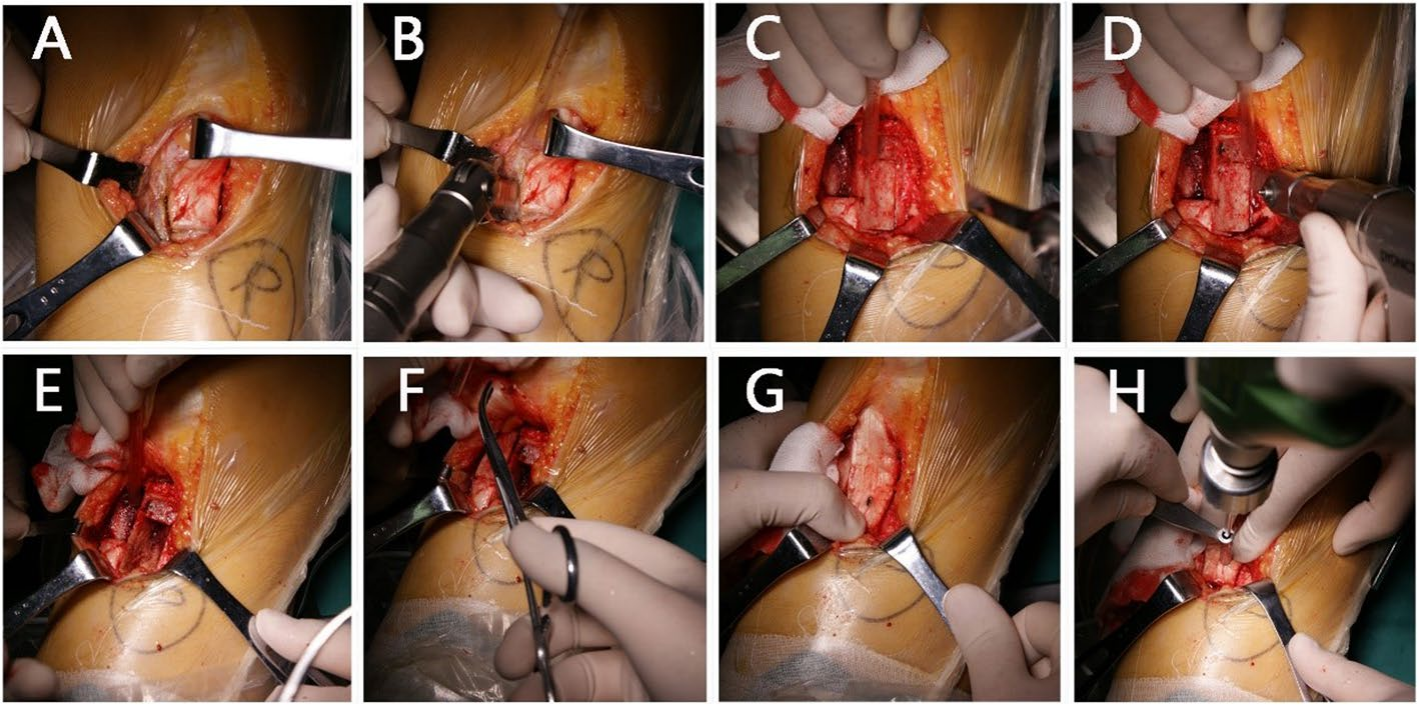
**A**-**B**: wedge-shaped osteotomy was done at the tibial tubercle; **C**- **D**: wedge-shaped osteotomy was done at the medial or /and inferior part of tibial tubercle; **E**–**G**: the position of the two bone mass was exchanged; **H**: the bone mass with patellar tendon was fixed with two 4.5 * 40 mm absorbable screws

**Fig. 4:**
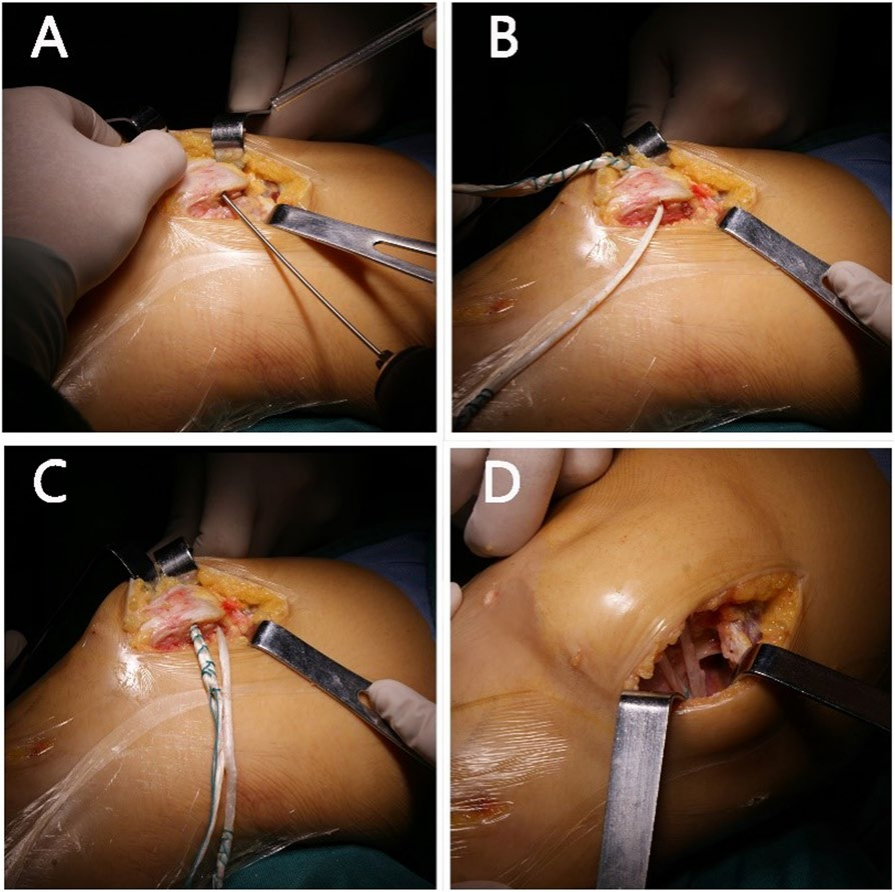
**A**: two parallel bone tunnels with a diameter of 4.5 mm were drilled in the medial side of the patella; **B**-**C**: The grafts were placed into the bone tunnel guided by a trans-femoral pin; **D**: another tunnel with a diameter of 6.0 mm was drilled on the site between the adductor tubercle and the proximal part of the superficial medial collateral ligament, the graft was then secured within the tunnel with a diameter of 6-mm interference screw and fixed at 30° of knee flexion

All surgical procedures were performed by one senior surgeon, who used the same technique. The patient was placed in supine position with a tourniquet placed on the thigh root. An autogenous hamstring tendon graft was then harvested and prepared. A 12-cm longitudinal incision was made on the medial side of patella. The patella was pushed toward the medial side. If the patella moved less than one fourth of the patella, the lateral patellar retinaculum would be partially severed for the release. The range of release was determined by the patella movement. A continuous incision which went through the capsule and lateral retinaculum ranging from, and including the vastus lateralis tendon to the joint line, 1 cm lateral to the patella was usually made. After the lateral retinaculum was released, the periosteum was dissected with osteotome at the medial or /and inferior part of tibial tubercle. Then wedge-shaped osteotomy was done at the medial or /and inferior part of tibial tubercle.The area of the bone mass was about 3.0 * 1.0 cm. After the wedge-shaped osteotomy, another wedge-shaped osteotomy was done at the tibial tubercle. The area of the bone mass with patellar tendon was about 3.0 * 1.0 cm. Then the position of the two bone mass was exchanged. The bone mass with patellar tendon was fixed with two 4.5 * 40 mm absorbable screws. After the transferring of the tibial tubercle, two parallel bone tunnels with a diameter of 4.5 mm were drilled in the medial side of the patella, and another tunnel with a diameter of 6.0 mm was drilled on the site between the adductor tubercle and the proximal part of the superficial medial collateral ligament. The grafts were placed into the bone tunnel guided by a trans-femoral pin. Appropriate graft tension was obtained by cycling the knee from full extension to full flexion. The graft was then secured within the tunnel with a diameter of 6-mm interference screw and fixed at 30° of knee flexion. The detail was seen in Figs. 3 and 4

### Postoperative Functional Exercise

A quality post-operative rehabilitation program was essential to having a successful outcome from a patellar stabilization procedure. In early stage, the goals of rehabilitation would initially focus on protection for healing, mobility and range of motion. Twenty-four hours after operation, ankle pump and straight-leg raise started with the knee joint fixed in extension position for two weeks. Then the knee flexion exercise started. At week 6, the knee flexed to 120°. The patients returned to daily activities around the third month.

### Statistical analysis

All calculations were made with IBM SPSS 25.0. Statistical significance was set at P < 0.05. Number of observed values, mean and SD, and minimum and maximum were reported for quantitative data. Number of observed values and number and percentage of patients per class were reported for qualitative data. Wilcoxon sign ranked sums was used for evaluating continuous variables and χ-square analysis was used for evaluating categorical variables.

## Results

### Population

A total of 108 knees in 98 patients were included in the study. Of these, 10 patients (10.2%) had bilateral reconstructions. In the follow-up, 15 patients (13.3%) were lost. The mean age at operation was 19.2 ± 6.1 years (range, 13–40 years), and the mean follow-up was 61.3 ± 15.4 months (range, 36–92 months). All patients included had trochlear dysplasia (A, 24%; B, 17%; C, 35%; D, 24%), and 67% had patellar alta. The mean CDI was 1.2 (range, 0.5–1.6); mean TT-TG distance, 22 mm (range, 17–30 mm); All data details were summarized in Table 1.

**Table 1:** Descriptive Characteristics of the Population before Surgery

Variable	n(%)
Sex	
male	30 (28)
female	78 (72)
Age at surgery, y	
Mean ± SD	19.2 ± 6.1
Minimum; Maximum	13; 40
Body mass index, kg/m^2^	
Mean ± SD	21.7 ± 3.4
Minimum; Maximum	16.6; 37.6
Side	
Right	52 (48)
Left	56 (52)
Patellar height(CDI)	
Mean ± SD	1.2 ± 0.22
Minimum; Maximum	0.5; 1.6
Class of patellar height	
< 1.3	36 (33)
≥ 1.3	72 (67)
Trochlear dysplasia	
Type A	26 (24)
Type B	18 (17)
Type C	38 (35)
Type D	26 (24)
TT-TG distance, mm	
Mean ± SD	22 ± 3.0
Minimum; Maximum	17; 30
Class of TT-TG distance, mm	
< 20	30 (28)
≥ 20	78 (72)
Postoperative Kujala score	
Mean ± SD	90.5 ± 15.5
Minimum; Maximum	24; 100
Postoperative Tegner score	
Mean ± SD	5.3 ± 1.3
Minimum; Maximum	2; 8
Postoperative IKDC score	
Mean ± SD	72.7 ± 12.1
Minimum; Maximum	26; 86
Follow-up, m	
Mean ± SD	61.3 ± 15.4
Minimum; Maximum	36; 92
Status at the follow-up	
Success	102 (94)
Failure	6 (6)

### Postoperative Outcomes

**Fig. 5:**
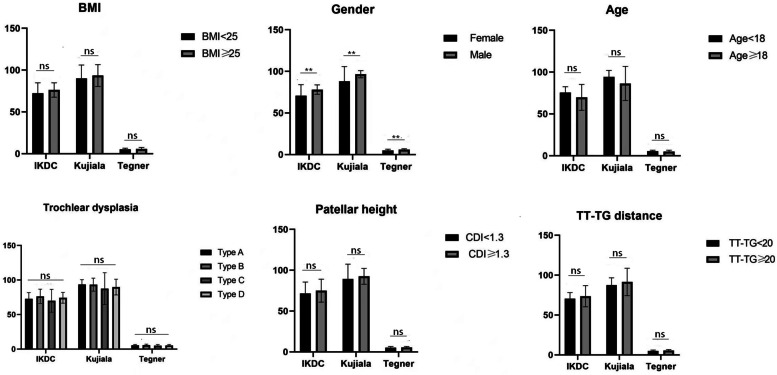
Outcomes According to Risk Factor

Six knees (5.6%) had postoperative dislocation, 4 with medial subluxation, 4 with lateral subluxation and 4 with obvious knee pain (VAS ≥ 4), 2 with operative incision disruption, 2 with fat liquefaction. All of the TTOs were clinically and radiographically healed by 3 months. The mean scores for Kujala, Tegner and IKDC were 90.5 ± 15.5 (range, 24–100), 5.3 ± 1.3 (range, 2–8) and 72.7 ± 12.1 (range, 26–86), respectively. Patients with grade-A trochlear dysplasia had mean Kujala, Tegner and IKDC scores of 93.6, 5.5 and 72.8. Patients with grade-B trochlear dysplasia had mean Kujala, Tegner and IKDC scores of 93.3, 5.8 and 76.4. Patients with grade-C trochlear dysplasia had mean Kujala, Tegner and IKDC scores of 87.5, 5.1 and 70. Patients with grade-D trochlear dysplasia had mean Kujala, Tegner and IKDC scores of 89.8, 5.3 and 74.2. There were no significant differences in postoperative outcomes for different grades of trochlear dysplasia (IKDC: *p* = 0.587; Tegner: *p* = 0.542; Kujala: *p* = 0.542). Patients with patellar height ≥ 1.3 had mean Kujala, Tegner and IKDC scores of 92.5, 5.4 and 75 as compared with 89.4, 5.3 and 71.5 for those with patellar height < 1.3 (IKDC: *p* = 0.329; Tegner: *p* = 0.882; Kujala: *p* = 0.458). Patients with TT-TG ≥ 20 mm had mean Kujala, Tegner and IKDC scores of 91.5, 5.4 and 73.6 as compared with 87.6, 5.1 and 70.4 for those with TT-TG < 20 mm (IKDC: *p* = 0.398; Tegner: *p* = 0.516; Kujala: *p* = 0.418). Patients with age ≥ 18 had mean Kujala, Tegner and IKDC scores of 86.4, 5.1 and 69.8 as compared with 94.3, 5.6 and 75.5 for those with age < 18 (IKDC: *p* = 0.398; Tegner: *p* = 0.516; Kujala: *p* = 0.418).Patients with BMI ≥ 25 had mean Kujala, Tegner and IKDC scores of 86.4, 5.1 and 69.8 as compared with 94.3, 5.6 and 75.5 for those with age < 25 (IKDC: *p* = 0.468; Tegner: *p* = 0.307; Kujala: *p* = 0.621).Female patients had mean Kujala, Tegner and IKDC scores of 88.2, 5.1 and 70.7 as compared with 96.6, 6.0 and 78.1 for male patients (IKDC: *p* = 0.006; Tegner: *p* = 0.006; Kujala: *p* = 0.008).The results showed that increasing trochlear dysplasia, increasing patellar height,, increasing TT-TG distance, BMI and age did not have any significant impact in the improvement of their Kujala, Tegner and IKDC scores, but Female gender as risk factor had a negative impact for IKDC, Tegner and Kujala scores. All data details were summarized in Fig. 5.

### Failure Risk Factors

The χ-square analysis indicated that there were significant differences in the failure rates among different grades of trochlear dysplasia (*p* = 0.016), and between age < 18 and age ≥ 18 (*p* = 0.011). The result showed that the failure rate was significantly associated with trochlear dysplasia and age. However, there were no significant differences in the failure rates between CDI ≥ 1.3 and CDI < 1.3 (*p* = 0.088), between females and males (*p* = 0.669), between BMI < 25 and BMI ≥ 25 (*p* = 1.000) and between TT-TG < 20 and TT-TG ≥ 20 (*p* = 0.336). The result showed that the failure rate was not significantly associated with patellar height, sex, BMI and TT-TG distance. All data details were summarized in Table 2.

**Table 2:** Analysis of Preoperative Failure Risk Factors of Combined Treatment

Analyzed Factor	patients, n	Comparison	*P* value
Patellar height (CDI)	108	≥ 1.3 vs < 1.3	0.088
Sex	108	female vs male	0.669
Age, y	108	≥ 18 vs < 18	0.011
BMI, kg/m^2^	108	≥ 25 vs < 25	1.000
TT-TG distance, mm	108	≥ 20 vs < 20	0.336
Trochlear dysplasia	108	type A vs B vs C vs D	0.016

## Discussion

The purpose of this study was to report the clinical outcomes of MPFL reconstruction combined with LRR and TTO in cases of patellar instability and to identify predictive risk factors for failure. The main finding was that the combined treatment was reliable with low failure rate. This study indicated that female gender was a risk factor for lower postoperative outcomes. Age and trochlear dysplasia were considered to be two failure factors for MPFL combined with TTO and LRR. What is more, if the patellar was pushed toward less than one fourth of the patellar width, the release of lateral retinaculum was suitable. If TT-TG distance ≥ 20 mm, the amount of medialization-10 mm was reliable.

Previous studies showed that above 90% of the patients had trochlear dysplasia [14–16]. In the current study cohort, all the patients had trochlear dysplasia. However, the proportion should be below 100% considering the loss to follow-up. The Kujala score with the scale from 0 to 100 was most frequently utilized for measuring patient-reported outcome among patellofemoral studies [13]. The postoperative outcome was in a ‘‘good’’ category when a score ranged from 85 to 94.31. The mean Kujala score was 90.5 which should be considered good outcome. The mean Kujala scores of trochlear dysplasia A, B, C and D were 93.6, 93.3, 87.5 and 89.8, respectively. There were no significant differences among different grades. This result demonstrates that the combined treatment can be successful and should be suitable for patients with severe trochlear dysplasia. Allen et al. [7] also reported that the combination of MPFL reconstruction and TTO in patients with trochlear dysplasia results in low recurrence of instability and good subjective outcomes.

In terms of anatomical structure, the slope of the lateral trochlear facet keeps the patellar from lateral displacement [17]. A flattened trochlear groove reduced lateral stability by 70% at 30 of flexion Thus, in the treatment of recurrent lateral patellar dislocation, trochleoplasty is utilized in cases of severe trochlear dysplasia. Several studies indicated that combined MPFL reconstruction with trochleoplasty could result in good postoperative stability and patient satisfaction [18, 19]. However, recently many concerns were raised on the complication rate and the risk to the cartilage and progress of arthritis in the patellofemoral joint after this procedure [20]. In addition, the procedure is technically demanding. Trochlear dysplasia is a common finding among patients with recurrent patellar instability, which might lead to patellofemoral maltracking and especially the presence of a positive J-sign at the clinical examination. One research indicated that a significant correlation existed between a positive J-sign and severe trochlear dysplasia and a positive J-sign was a risk factor for failure. However, trochlear dysplasia did not reliably predict the risk of failure [16]. This inconsistency might be related to the Dejour classification’s fair intra- and inter-observer reliability. Trochlear dysplasia might be a risk factor of failure. In our study cohort, there were significant differences in the failure rates among different grades of trochlear dysplasia (p = 0.016), which indicated, trochlear dysplasia could predict the risk of failure. Thus, trochleoplasty as a treatment should be considered in cases of severe trochlear dysplasia.

When the patellar moves through the sulcus; its routine is not straight. When the knee is in full extension, the patella lies on the superolateral side of the femoral sulcus. When the knee is in10 to 30° of flexion, the engagement between the patella and trochlea occurs. The relation can be affected by changes in patellar tendon length. For patients with patella alta, the engagement between the patella and trochlea happens at greater flexion angles, leading to less bony constraint at earlier degrees of flexion [21]. Thus, in the treatment of recurrent lateral patellar dislocation, patella alta is an indication for the distalization of the tibial tubercle. A study with the largest sample size for isolated MPFL reconstruction for recurrent patellar instability indicated that CDI > 1.3 was one preoperative failure risk factor. In our study for combined treatment, patellar alta was not a failure risk factor any more due to the distalization of the tibial tubercle. Thus, TTO for patellar alta was necessary for patellofemoral stability.

TT-TG-a distance between the tibial tuberosity and the trochlear groove-exceeding 20 mm is nearly always associated with patellar instability. Patients with TT-TG > 20 mm are always recommended with TTO treatment. Several studies indicated isolated MPFL construction for patients with increased TT-TG resulted in lower postoperative outcomes and subsequent instability [22, 23]. In addition, Stephen et al. [24] proved that isolated MPFL construction would add the graft tension and resulted in degenerative changes of the graft. Thus, medialization of the tibial tubercle was necessary for patellofemoral stability. The suitable extent of medialization varied; however, postoperative TT-TG distance from 9 to 15 mm was proposed by the majority of researchers [25, 26]. The over-medializing of the tibial tubercle could increase contact pressure and cause pain [27]. In our study, four patients were with medial subluxation. Thus, Care should be taken to assess the patellar tracking within the trochlear groove for avoiding the over-medializing of the tibial tubercle.

In our study, females were observed to have worse clinical scores. There were no significant differences in age, BMI, patellar alta, trochlear dysplasia, and TT-TG between females and males which was also proved by Allen et al. [7]. And several studies also proved that there were a few risk factors for recurrent patellar inability including ligamentous laxity, rotational abnormalities and higher Q angle [28, 29]. Our findings support that sex is an important factor in the postoperative outcomes. Besides trochlear dysplasia, age was observed to be a risk factor for failure. Bone grows rapidly in the period of adolescence [30]. The morphological structure of immature bone is highly responsive to its contact environment, because mechanical forces influence both osteogenesis and bone remodeling [31–33]. Improvement of patellar alignment at an early age maybe promotes better further development of the anatomic relationship between the patella and femoral trochlear groove, and on earth make patella more stable [34]. Considering the risk of growth arrest and recurvatum deformity, skeletal immaturity with an open tibial apophysis is a strict contraindication for the osteotomy. Thus, the patients younger than fourteen years old is not advised to accept the osteotomy [35, 36]. In our study, the minimum age for combined surgery was 13 years old. No patients showed up growth arrest and recurvatum deformity. The mean postoperative Kujala score of patients younger than eighteen years old was 91 which was in good category. And no recurrent dislocation happened in patients younger than eighteen years old.

Lateral retinacular release is a useful treatment for patellar inability. The imbalance of the extension mechanism caused by excessive tension of the lateral retinaculum always results in patellofemoral disorders. Although the contribution of the lateral retinaculum to the lateral stability is only 10%, it often leads to abnormal contact between the lateral surface and the trochlea under excessive tension and therefore patellar mal-tracking [37]. In patellar dislocation, medial patellar ligament injury and quadriceps femoris weakness often occur, which leads to lateral collateral ligament contracture and excessive tension. We believed that proper lateral retinaculum release was necessary to maintain the stability of patella. The main complication of lateral retinaculum release was patellar medial dislocation or subluxation, but the incidence rate was very small. This was also confirmed in this study where Only 4 patients had medial patellar subluxation.

This study had several limitations. First, this study was a retrospective analysis of patellar inability. Second, 15 patients (13.3%) were lost to follow-up despite attempts to contact them by telephone, which might cause bias risk. Third, among the patients contacted for the follow-up, no patients completed the preoperative assessment of the function of knee joint using related scales, which make us unable to evaluate the difference between preoperative and postoperative. Nevertheless, this was the first study about the function of combined treatment (MPFL reconstruction, tibial tubercle osteotomy and lateral retinacular release) at least three years after surgery and preoperative risk factors.

## Conclusion

As a surgical method, MPFL combined with TTO and LRR would be a reliable choice with a low failure rate (5.6%). Female gender was a risk factor for worse postoperative outcomes. Preoperative failure risk factors in this study were age and trochlear dysplasia.

## Data Availability

The datasets supporting the conclusions of this article are included within the article. Raw data can be requested from the corresponding author.

## Abbreviations

MPFL: Medial patellofemoral ligament
TTO: Tibial tubercle osteotomy
LRR: Lateral retinacular release
CDI: Caton-Deschamps index
TT–TG: Tibial tuberosity to trochlear groove
TT-PCL: Tibial tuberosity to posterior cruciate ligament
CT: Computed tomography
BMI: Body mass index
